# A clinical study of relationship between obesity and pubertal development in girls

**DOI:** 10.1186/1687-9856-2013-S1-P76

**Published:** 2013-10-03

**Authors:** Phil Soo Oh, Hui Kwon Kim, Jeh Hoon Shin

**Affiliations:** 1 Department of Pediatrics, Hallym University Medical Center, Chuncheon, Korea; 2 Department of Pediatrics, Hanyang University Medical Center, Seoul, Korea

## Purpose

Recently, public interests for obesity and earlier pubertal development has been increasing. The purpose of this study was to analyze the relationship between obesity and pubertal development in girls.

## Methods

This study was performed for 158 girls with earlier pubertal development from July 2008 to June 2010. Their mean age was 8.27±1.3 years and mean bone age advancement was 1.86±0.3 years.

## Results

1. Upon the weight-for-height percentiles, their obesity rate was 9.2% and overweight rate 15.8%. However, upon BMI the result was a little different, that is, their obesity rate is 13.2% and overweight rate is 24.3%. 2. About 40% of the girls had a family history of early maturation. Among them, 25.7% had a maternal history, 4.6% paternal and 7.2% both. However, 60% of them had no family history. 3. We then classified these girls into the families with one daughter and one son and those with two daughters. In one-daughter/one-son families, 65.3% were the first children and 30.8% were seconds. In two-daughters families, 65.3% were the firsts and 34.7% were seconds. 4. We found that 67.5% had a history of taking herbal medicine materials.

## Conclusions

The obesity and overweight rate in girls with earlier pubertal development was higher than the age-matched normal females, but with a little discrepancy between weight-for-height percentile and BMI-based data. And, it seems to be possible that herbal medicine materials is a potential factor for earlier pubertal development in Korea.

